# The effect of platelet-rich plasma on the fusion rate and clinical outcome of spinal fusion surgery: A systematic review and meta-analysis

**DOI:** 10.1371/journal.pone.0243204

**Published:** 2020-12-03

**Authors:** Ying-feng Cai, Tian-Zhao Tian, Li-Ye Chen, Bao-Xin Liu, Jian-Peng Zhou, Min Shi, Hao-Dong Liang

**Affiliations:** Department of Orthopaedics, The Affiliated TCM Hospital of Guangzhou Medical University, Guangzhou, People's Republic of China; University of Camerino, ITALY

## Abstract

**Background:**

Platelet-rich plasma (PRP) is widely used in many orthopedic surgeries and spinal disease treatments; however, the effect of PRP on spinal fusion remains controversial.

**Questions/Purposes:**

To assess the fusion rate and clinical results of PRP compared with non-PRP administration in the treatment of spinal fusion with regard to decreasing pain and improving healing and function.

**Patients and methods:**

Studies comparing PRP to non-PRP treatment with respect to the fusion rate and clinical outcome in patients who underwent spinal fusion surgery were included.

**Result:**

Three randomized controlled trials (RCTs) and 7 prospective cohort studies were identified. The spinal fusion rate was not significantly different between the groups in all RCTs or cohort studies at the final follow-up. In comparison, PRP significantly reduced pain after surgery as evaluated in the RCT analysis and the complication rate did not differ significantly between the two groups.

**Conclusion:**

According to the available studies, PRP does not contribute to the union rate, relieve pain or increase the complication rate in spinal fusion surgery. As clinical heterogeneity exists in these studies, further large, well-designed RCTs that focus on the standard assessment of PRP are needed.

## Introduction

Spinal fusion is an effective treatment method for spinal diseases, such as disc herniation, spinal instability and degenerative spinal diseases. However, studies have reported 5%-43% nonunion and pseudarthrosis after spinal fusion surgery [1, 2], which can lead to poor clinical outcomes and increase the economic burden of patients. Therefore, many strategies have been created to accelerate bone union [3, 4].

Treatment with platelet-rich plasma (PRP) is a promising strategy that uses a small amount of plasma containing a high concentration of platelets and osteoinductive autologous growth factors and has been reported to help the regeneration of bone and tissue. The additional platelets may substantially increase the concentration of growth factors at the site of injury and augment the natural healing process [5]. Studies have shown the efficacy of accelerating bone union and spinal fusion in vivo and in vitro. PRP has also been used in ligament reconstruction surgeries to enhance ligament and articular regeneration and simultaneously relieve pain. Animal and clinical studies have also suggested a positive effect of PRP [6–8].

With the increasing attention to and wide use of PRP in orthopedics, there is a growing debate regarding its clinical efficacy, especially in spinal surgery. Some randomized controlled trials (RCTs) have shown less favorable results for the fusion rate and pain score [8, 9], which were in contrast to the results of other studies. Given this uncertainty, we undertook a systematic review and meta-analysis of RCTs and cohort studies to assess the fusion rate and clinical results of PRP compared with non-PRP in the treatment of spinal fusion, with regard to decreasing pain and improving healing and function.

## Methods

### Eligibility criteria

Studies comparing PRP to non-PRP with respect to the fusion rate and clinical outcome in patients who underwent spinal fusion surgery were included. Inclusion criteria were defined before the search process, and are listed as follows: (1) patients with degenerative disc diseases, lumbar disc herniation, spinal instability and other spinal diseases who were scheduled for spinal fusion; (2) RCTs and cohort studies comparing PRP with non-PRP; and (3) primary outcomes that include fusion rate and adverse events related to PRP and the visual analog scale (VAS). The secondary outcomes included the clinical outcome assessed after surgery, such as the patient report Short Form 36 (SF-36) and the Oswestry Disability Index (ODI). Clinical trials without comparison groups or animal studies or laboratory research were excluded form our research.

### Search strategy

The methods in this review were conducted in accordance with the Cochrane Collaboration criteria [10]. A comprehensive literature search of relevant RCTs and cohort studies involving both PRP and spinal fusion was performed in PubMed, Embase, and the Cochrane Library from database inception to April 2019. Database searching was performed using the following terms: “spinal fusion,” “spondylodesis,” “spinal arthrodesis,” “Lumbar arthrodesis,” “platelet-rich plasma,” “platelet gel,” “platelet fibrin,” “platelet glue,” and “PRP”. The full details of the search process are shown in S1 Table.

The main search was completed by the senior authors (YFC and LYC). Any discrepancy was identified by discussion with another investigator (HDL) who was not involved in the initial procedure. The reference lists of eligible studies and ‘‘related articles” featured in PubMed were also reviewed in our search. The ages of the patients and follow-up periods were not restricted, and the publication language was limited to English. If more than one study was identified from the same population, data from the most recent or complete report of that study were extracted.

#### Study selection and data extraction

Two investigators (YFC, TZT) independently extracted the relevant data from each study using a predefined data extraction form listing the study characteristics, which included the first author’s name, publication year, country, study design, follow-up duration and outcome measurements for each study. Any disagreements were resolved by discussion or consensus with a third reviewer (BXL). Radiological fusion was defined as the absence of significant angular motion (no more than 2˚) on flexion and extension radiographs and evidence of bridging bone on computed tomography (CT) scans or plain graphs [11]. The fusion rate was calculated by the number of patients who achieved fusion divided by the total cohort. Complication rates were calculated on the basis of events related to surgery, such as pseudarthrosis, infection, reoperation and seroma, according to previous studies [12, 13]. Specific complications, such as donor site pain, were not included in our analysis. The corresponding authors of the included RCTs were contacted to obtain any missing data when necessary.

#### Outcome and methodological quality assessment

The methodological quality and risk of bias of the included studies, including randomization, allocation concealment, blinding method, selective reporting, group similarity at baseline, incomplete outcome data, compliance, timing of outcome assessments, and intention-to-treat analysis, were separately assessed by two reviewers (YFC and BXL) using the Cochrane Collaboration tool [10]. All disparities between the two reviewers were resolved by consultation with another author (LYC). A quality assessment of observational studies was conducted in accordance with a modified version of the Newcastle-Ottawa Quality Assessment Scale (NOS) [14]. A maximum of two stars was given for comparability. We obtained 60% or more of the maximum number of stars as high-quality studies [14].

#### Statistical analysis and data synthesis

Pooled analyses were performed using Review Manager (RevMan Version 5.3., the Cochrane Collaboration, Oxford, UK). Risk ratios (RRs) with a 95% confidence interval (CI) were used to assess dichotomous outcomes. Continuous outcomes were expressed as the mean difference (MD) with 95% CI. Heterogeneity was assessed using the I^2^ statistic. According to the Cochrane review guidelines, severe heterogeneity of I^2^≥50% warrants the use of a random-effects model. Otherwise, a fixed-effects model should be used. I^2^≥50% represented high heterogeneity. Subgroup analyses were performed according to different timepoints.

#### Ethics statement

The study was approved by the Ethics Committee of the Affiliated TCM Hospital of Guangzhou Medical University. This review was registered in PROSPERO: CRD 42019132218 (https://www.crd.york.ac.uk/PROSPERO/). The work was performed at the Affiliated TCM Hospital of Guangzhou Medical University.

#### Patient and public involvement

No patients or public parties were involved in this study.

## Results

### Literature search

**Fig 1:**
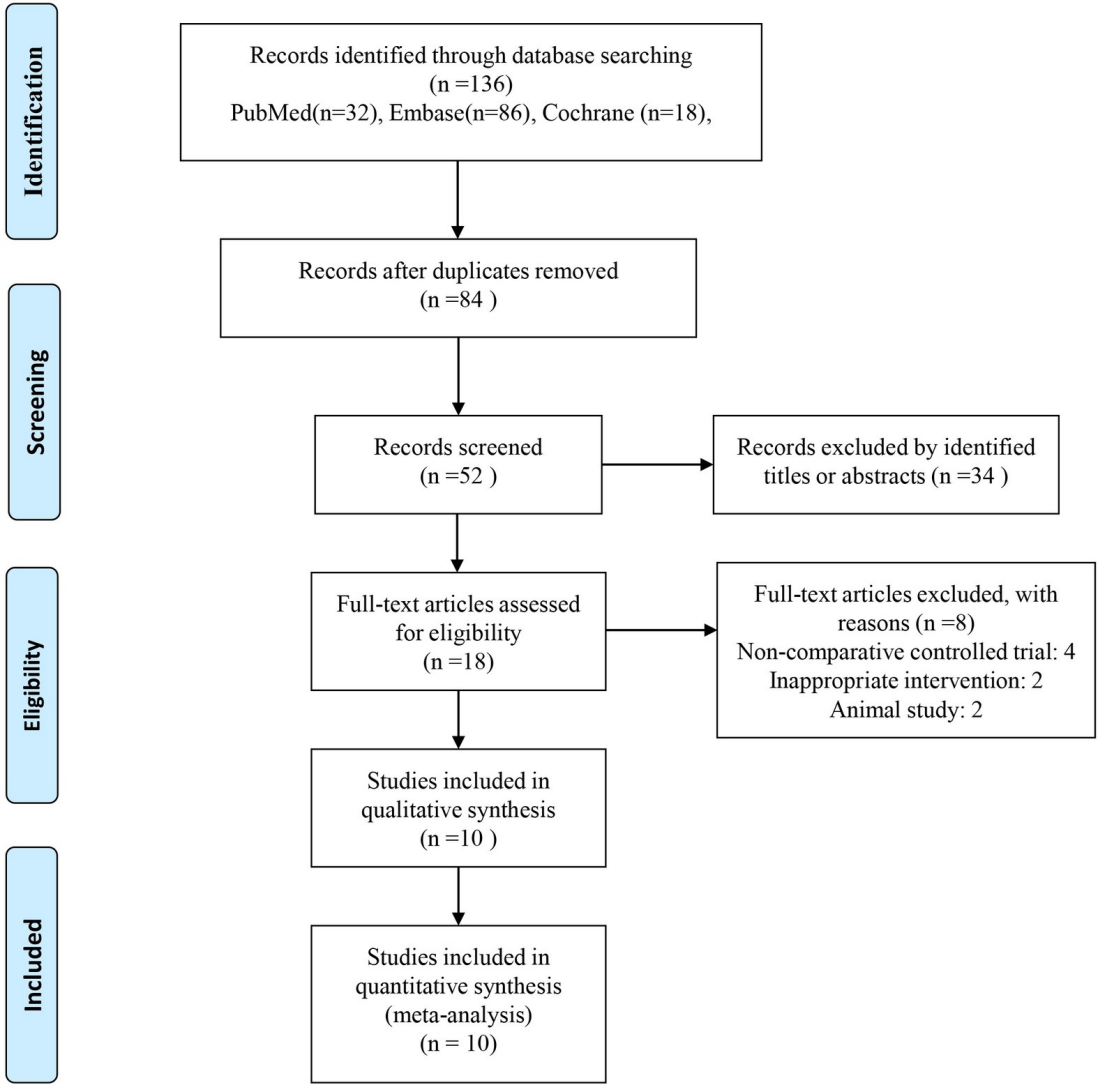
Flow diagram of the relevant study selection process.

We generated 136 relevant studies through a comprehensive search (Fig 1). After removing 84 complicated duplicate studies, 52 studies were retrieved. After screening the titles and abstracts of the remaining studies, 34 studies of the following types were excluded: in vivo or in vitro investigations (9), laboratory research (7), and studies on unrelated topics (3). We finally identified 18 potentially relevant studies. After careful full-text screening, the following publication types were excluded: noncomparative trial (4), inappropriate intervention (2) and animal studies (2). Three RCTs and 7 cohort studies published from 2003 to 2019 involving 3 different PRP types and 566 participants were ultimately included in the present study. The characteristics of the included studies are presented in Table 1. The total sample sizes were 69 for the PRP group and 69 for the control group in the RCTs and 202 for the PRP group and 226 for the control group in the cohort studies. The populations of the included studies ranged from 35 to 107 participants. The follow-up durations ranged from 8 months to 10 years.

**Table 1:** Characteristics of included studies.

Study/year		PRP Group/Control Group							
	Type of study	Number of participants	Number of levels	Age (Mean), y	Gender (% Male)	Follow-up (Mean), m	Surgical Indications	Surgical approach	Outcome assessment
Feiz-erfan/2007	RCT	25/25	42/39	46.0/46.0	NA	24/24	cervical degenerative disease and herniated disc	ACDFP	SF-36; NDI; VAS; modified Prolo scale; fusion rate
Sys/2011	RCT	19/19	19/19	74.9/76	63/24	24/24	lytic and degenerative spondylolisthesis, disc degeneration and herniated disc	posterior lumbar interbody fusion	VAS, ODI; SF-36; fusion rate; blood loss
Kubota/2019	RCT	25/25	Single:18/15; Two:7/10	65.1/65.3	60/56	24/24	lumbar spinal stenosis with spondylolisthesis plus instability	posterolateral lumbar fusion	VAS; fusion rate; duration for bone union; quantification of platelet count and growth factor concentrations
Gastro/2004	Cohort study	22/62	Single:15/37;Two:7/25	47.0/49.0	27/34	34/41	degenerative disc disease; stenosis; pseudarthrosis; spondylolisthesis	transforaminal lumbar interbody fusion	fusion rate; operative times; estimated blood loss; postoperative drainage; transfusion; length of hospitalization; arthrodesis rate; platelet counts; complications
Jenis/2006	Cohort study	15/22	Single:5/12; Two:10/10	40.3/41.4	67/64	24.3/25.7	degenerative disc disease and spondylolisthesis	anterior-posterior interbody fusion	SF-36; ODI; VAS; fusion rate
Tsai/2009	Cohort study	34/33	34/33	59.8/63.3	32/18	28.5/27.6	spondylolisthesis	posterolateral lumbar fusion	fusion rate; postoperative drainage; clinical outcome (MacNab criteria)
Hartment/2009	Cohort study	15/20	Single:7/11; Two:8/8; Three:0/1	43.7/39.8	67/65	8.33/12.5	traumatic fracture of the lower thoracic or lumbar spine	posterior and anterior spinal fusion	fusion rate; VAS
Acebal-cortina/2011	Cohort study	67/40	Single:34/25; Two:33/12; Three:0/3	57.0/59.0	43/60	24/24	lumbar degenerative pathologies	posterolateral lumbar fusion	fusion rate;
Imagama/2017	Cohort study	29/29	29/29	63.3/63.3	38/38	240/240	degenerative lumbar disease	posterolateral lumbar fusion	fused bone area; Japanese Association Score; Time Course of Bone Absorption; bone density; fusion rate
Rezende/2017	Cohort study	20/20	NA	49.18	NA	24/24	hernia with instability; spondylolisthesis; hernia with stenosis	NA	fusion rate

SF-36: Short Form–36; RCT: randomized controlled trial; VAS: visual analog scale; ACDFP: anterior cervical discectomy with internal fixation and plating; ODI: Oswestry Disability Index; NDI: Neck Disability Index.

PRP is typically prepared from autologous whole blood through two centrifugation steps. First is a ‘‘separation” step, in which the red blood cells are separated out from the plasma and platelets through centrifugation. The plasma and platelet layers are then removed and a second centrifugation step, the ‘‘concentration” step, is performed to extract a platelet pellet [15]. The PRP preparation methods in the included studies varied (Table 2). Feiz-erfan and Sys et al. used the same platelet concentration system (DePuy Symphony system) [16]. Castro and Jenis et al. concentrated platelets through an ultrafiltration device. Hartmann et al. prepared PRP using a Biomet Gravitational Platelet Separation system. Three activation methods were measured in the included studies. Three studies used CaCl_2_ and thrombin for activation, while four studies used thrombin or CaCl_2_ only. Moreover, the activation method was not reported in 2 studies. For the formulation of the PRP, most studies reported the formulation as a PRP gel. The eventual formulation reported by Jenis and Acebal et al. was a platelet-rich solution. Tsai et al. mixed the obtained platelets with fresh frozen plasma and a calcium chloride combination was then added to local fragmented laminectomy bone to finally obtain the platelet glue. All researches collected PRP from autologous whole blood in participants except Tsai et al., who obtained platelet concentrate from the blood bank. Acebal et al. reported that autologous platelet concentrate harvesting was performed by taking 100 cc blood, but they did not mention the preparation method. Castro and Jenis et al. collected 1 unit of whole blood from the patients. Kubota and Imagama et al. collected a total of 400 mL of whole blood from each patient, and then separated and centrifuged the sample to collected the plasma. Hartmann et al. prepared PRP from 110 ml of patient blood. The final volume of PRP was not available in most studies. Only Kubota et al. reported that they finally generated 22 ml of PRP. Rezende et al. did not report the detailed preparation method or formulation of PRP [17].

**Table 2:** PRP formulation.

Studies	Preparation method	Activation method	Formulation
Feiz-erfan et al.	DePuy Symphony system	NA	Platelet gel
Sys et al.	DePuy Symphony system	CaCl_2_ and thrombin	Platelet gel
Kubota et al.	Hitachi Koki, CR7B3	CaCl_2_ and thrombin	Platelet gel
Castro et al.	Interpore cross ultraconcentrator	thrombin	Platelet gel
Jenis et al.	Centrifugation and ultraconcentrator	thrombin	Solution
Tsai et al.	Platelet concentrate from the blood bank	CaCl_2_ and thrombin	Platelet gel
Hartmann et al.	Biomet Gravitational Platelet Separation system	thrombin	Platelet gel
Acebal et al.	NA	NA	Solution
Imagama et al.	Kubota 9800 Centrifuge	calcium gluconate and thrombin	NA
Rezende et al.	NA	CaCl_2_	NA

### Study characteristics and quality of evidence

Details of the bias assessment of RCTs and cohort studies are shown in Table 3. All included RCTs were performed at a single center. Kubota et al. reported random sequence generation defined as having a low risk of bias, while the remaining trials did not. All RCTs were defined as having a low risk of detection bias because the outcomes were assessed separately by professors who were blinded to the research. Incomplete outcome data were observed in one trial [9], as there was no detailed description of the two drop-outs. This factor was judged as having a high risk of attribution bias. A high risk of reporting bias was found in the study by Feiz-erfan et al., as no direct comparison of clinical outcomes was conducted between groups at all timepoints. For the prospective cohort study, the methodological quality of five studies [18–22] was considered to be high on the basis of the NOS (Table 3). The main deficiency of the Hartmann et al. study was reporting bias related to the control group that was drawn from different sources, with an insufficient description of the outcome assessment and a short follow-up.

**Table 3:** Risk of bias in the included studies.

	Domains of randomized control studies							
Study	Random sequence generation	Allocation concealment	Blinding of participants	Blinding of personnel	Blinding of outcome assessment	Incomplete outcome data	Selective reporting	
Feiz-erfan et al.	unclear	unclear	low	low	low	low	high	
Sys et al.	unclear	low	unclear	unclear	low	high	low	
Kubota et al.	low	unclear	low	low	low	low	low	
	Domains of Cohort studies							
	Representativeness of the exposed cohort	Selection of the nonexposed cohort	Ascertainment of exposure	Demonstration outcome already	Comparability of cohorts	Assessment of outcome	Follow-up time	Adequacy of follow-up of cohorts
Gastro et al.	low	low	low	high	low	high	low	low
Jenis et al.	low	low	low	high	low	low	low	low
Tsai et al.	low	low	low	high	low	high	low	low
Hartmann et al.	low	high	low	low	high	high	low	high
Acebal et al.	low	low	low	high	low	low	low	low
Imagama et al	low	low	low	high	low	low	low	low
Rezende et al.	high	high	low	high	low	high	low	low

## Outcomes of the meta-analyses

### Primary outcomes

#### Fusion rate

**Fig 2:**
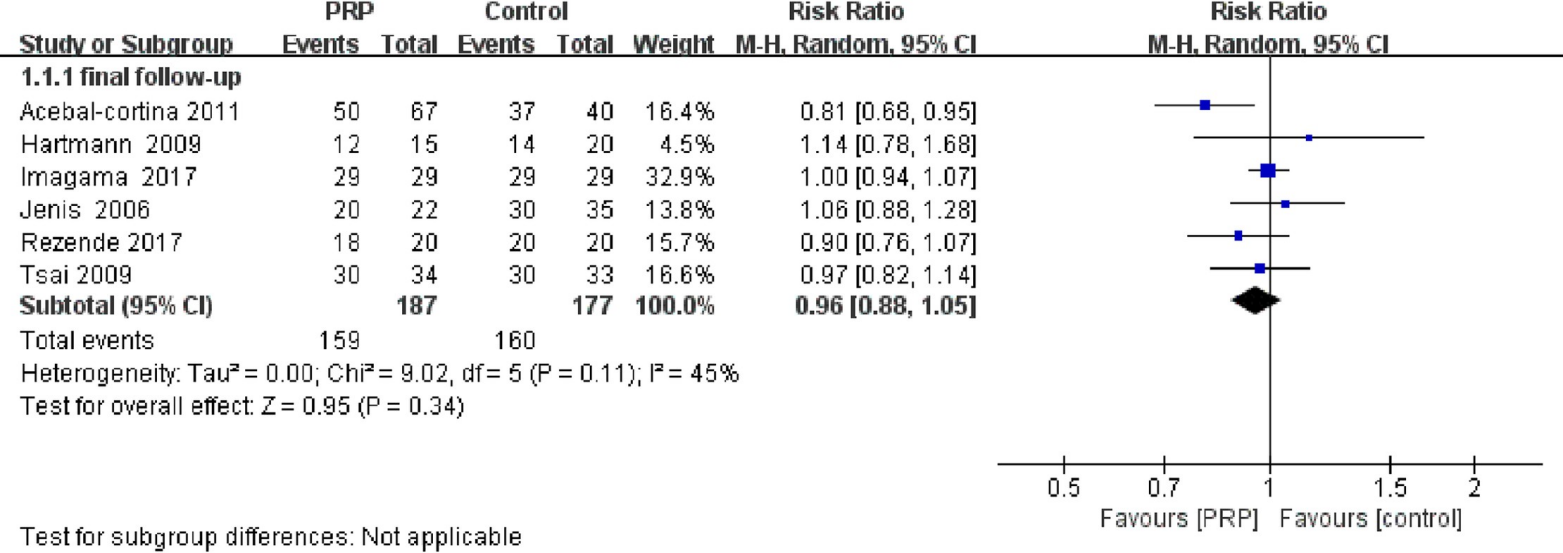
Forest plot of the fusion rate in cohort studies.

All of the included studies, which comprised 178 patients and 226 levels in RCTs and 388 participants and 408 levels in cohort studies, reported the fusion rate in spinal surgery. One study involved surgery on the cervical spine [16], and the other studies involved surgeries on the lumbar spine. The fusion rate was assessed at 3 months, 4 months, 6 months, 12 months, 24 months and 10 years postoperatively. Fusion was evaluated through CT in all studies, except four [16, 20, 23, 24], which were evaluated through X-ray imaging. Sys et al. reported that fusion was achieved in all but one patient from the control group, and no significant difference was found by CT scan between the two groups in interbody healing. Feiz-erfan et al. compared the fusion rate in patients with varied levels of surgery based on the levels of bony union and reported a higher fusion rate in the PRP group (18/30) than in the control group (12/25) at 6 and 12 weeks, but this difference was no longer apparent at one year. Kubota et al. assessed the bone union rate at two sites at each fusion level and showed a higher fusion rate in the PRP group than in the control group (93.7% vs 74.2%); the PRP group achieved a shorter time of bone union (7.8±0.6 vs 9.8±0.6 months). Data on the fusion rate and time needed for bony fusion in RCTs were not available for a pooled analysis because of the varied definitions and methods for evaluating fusion. A meta-analysis was performed in 6 cohort studies, as the study in which Castro et al. reported a fusion rate based on the fusion level was excluded from the present analysis. The pooled analysis did not show that PRP treatment was able to significantly increase the fusion rate in the final follow-up (RR = 0.96, 95% CI: 0.88–1.05, P = 0.11); however, there were no available data to perform a subgroup analysis evaluating the fusion rate at different timepoints postoperatively (Fig 2).

#### VAS

**Fig 3:**
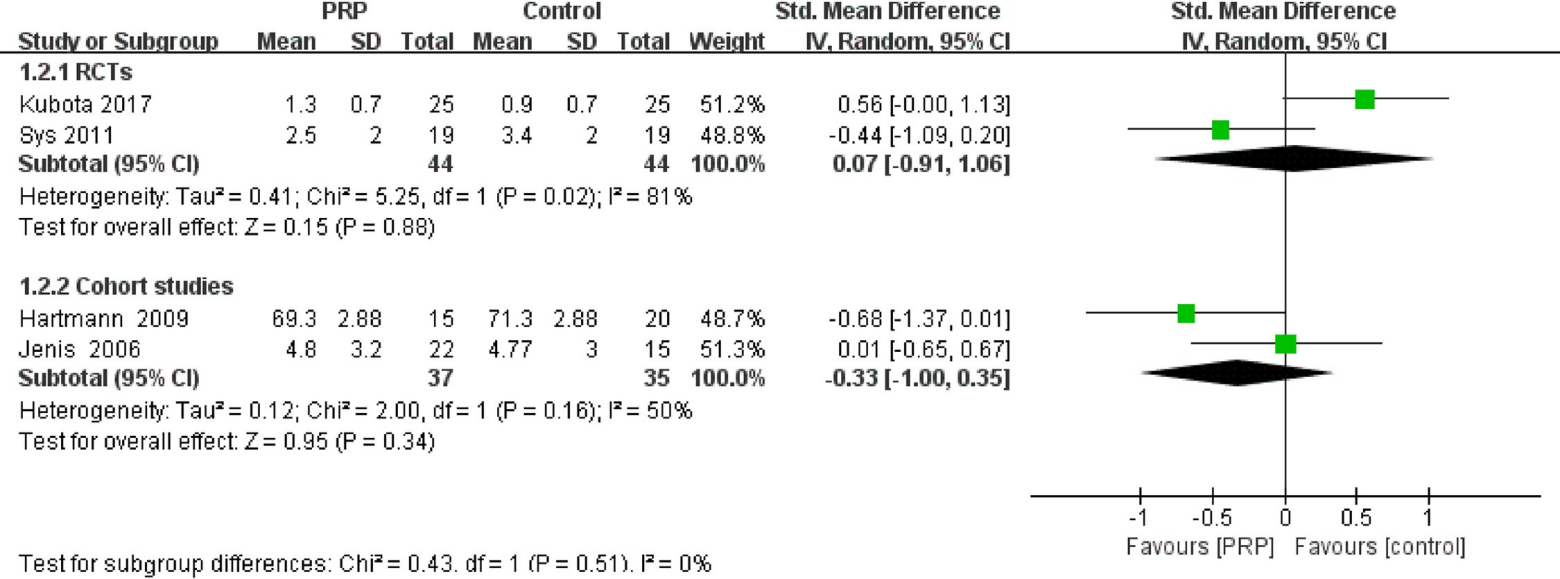
Forest plot of the VAS in the included studies.

All included RCTs assessed patients via the VAS; however, only two RCTs and two cohort studies compared the PRP group to the non-PRP group (Fig 3). The other study compared degenerative diseases with herniated disc diseases. The pooled analysis of RCTs showed that PRP relieved pain better than the control treatment, with a significant difference (SMD = -0.07, 95% CI: -0.91–1.06, P = 0.02). However, the pooled analysis of cohort studies suggested no significant difference between groups (SMD = -0.33, 95% CI: -1.00–0.35, P = 0.16).

#### Complications

**Fig 4:**
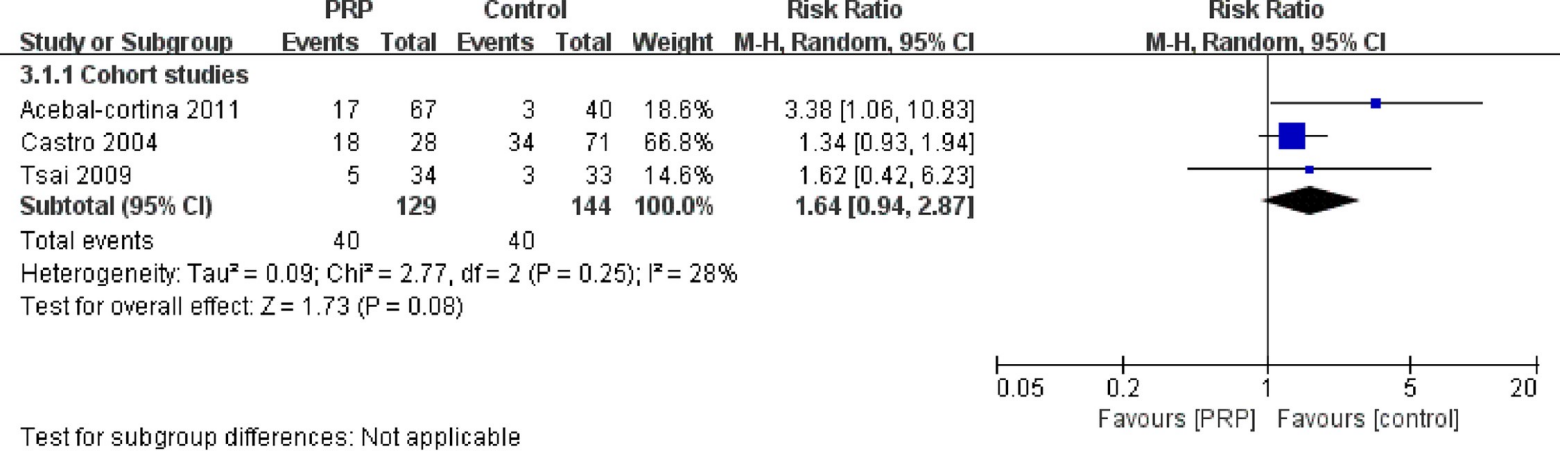
Forest plot of complications in the cohort studies.

Complications were varied and recorded in all of the included studies; such adverse events included pseudarthrosis, reoperation, infection, etc. No pseudarthrosis or other complications were reported in four studies [8, 19–21]. Sys et al. reported that transient radiculopathy occurred in one patient in the study group and two patients in the control group. Feiz-erfan et al. reported that a screw protruded minimally in two patients and that one patient died of cardiac disease. However, the details of the groups comprising these patients are not known. Jenis et al. reported 2 revisions, as one patient experienced vein injury and another experienced symptomatic pseudarthrosis. The complication rate was compared in only one study (by Castro et al.), and the results showed a lower complication rate in the study group than in the control group. These authors reported complications in 14 patients in the study group and 41 patients in the control group. Among these patients, 6 revisions in the control group were due to instrumentation malposition, and 5 revisions in the PRP group were due to pseudarthrosis. The other complications recorded in these studies were adjacent segment degeneration, arachnoiditis, intractable pain, dural tears, instrument malposition, instrument removal, infections and death. Meta-analyses were available for cohort studies (Fig 4), and the results suggested that the complication rate did not differ significantly between the two groups (RR = 1.19, 95% CI: 0.82–1.72, P = 0.38).

### Secondary outcomes

#### Pseudarthrosis

**Fig 5:**
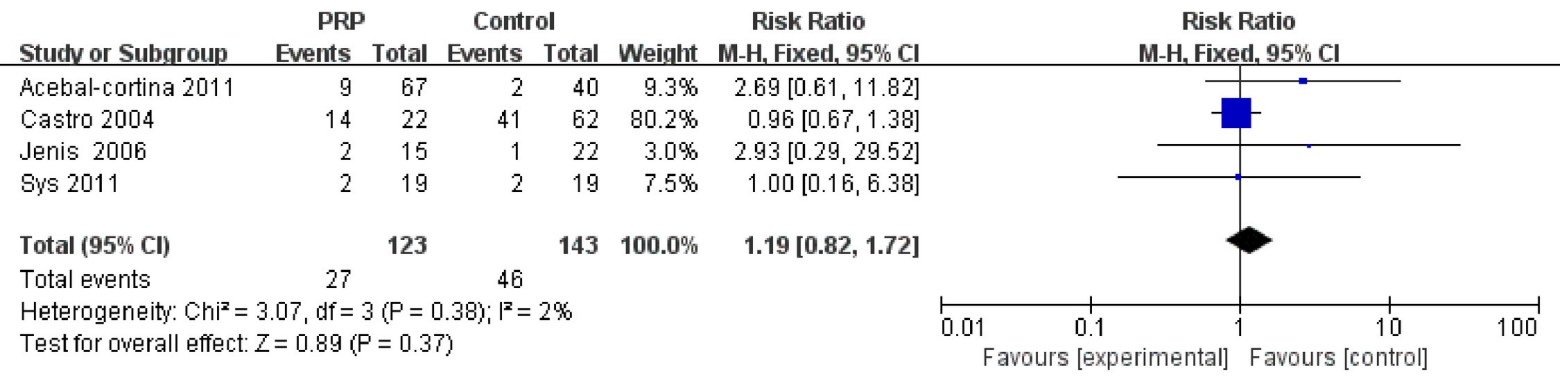
Forest plot of pseudarthrosis in the included studies.

Pseudarthrosis was reported in four studies. Assessment methods, including X-ray and CT, revealed that only one RCT [16] and three cohort studies reported comparisons of pseudarthrosis (Fig 5). The pooled analysis of cohort studies showed no significant difference between groups (RR = 1.64, 95% CI: 0.94–2.87, P = 0.25).

#### SF-36

Three studies assessed clinical outcomes based on SF-36 [9, 16, 22]. Feiz-erfan et al. compared the average score pre- and postoperatively, but there were no direct comparisons between groups. The overall mean score of SF-36 was increased at the 1-year and 2-year follow-up, and patients with herniated disc diseases had higher mean scores on the SF-36 than those with degenerative disc diseases. Both groups showed improvements in each domain of the SF-36 in the Sys et al. study. There were clinically important improvements in each domain (7.0 points) and statistically significant improvements in 6 domains; however, improvements in vitality and mental health in the PRP group were not statistically significant.

#### Publication bias

**Fig 6:**
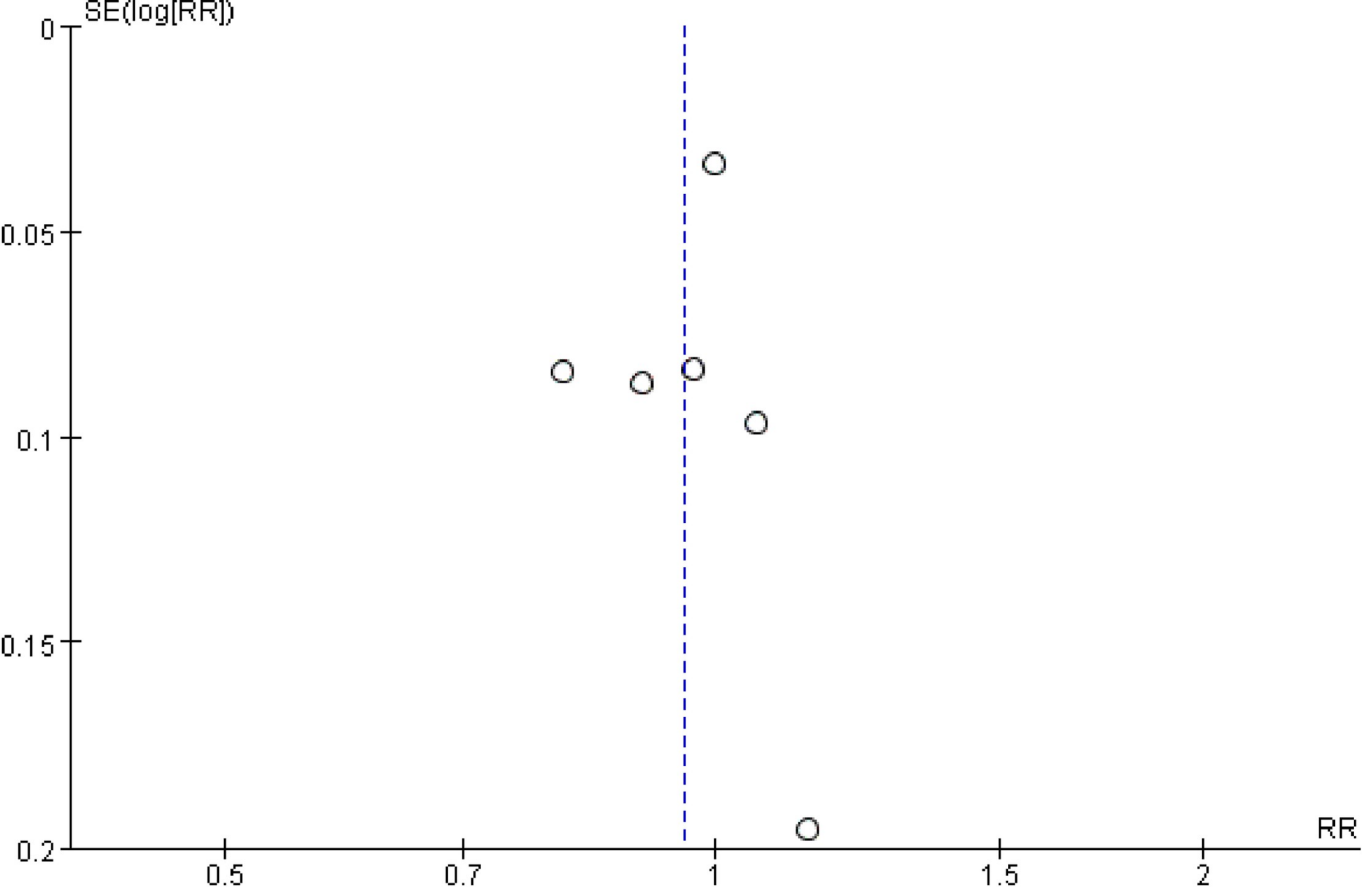
Funnel plot of publication bias.

**Fig 7:**
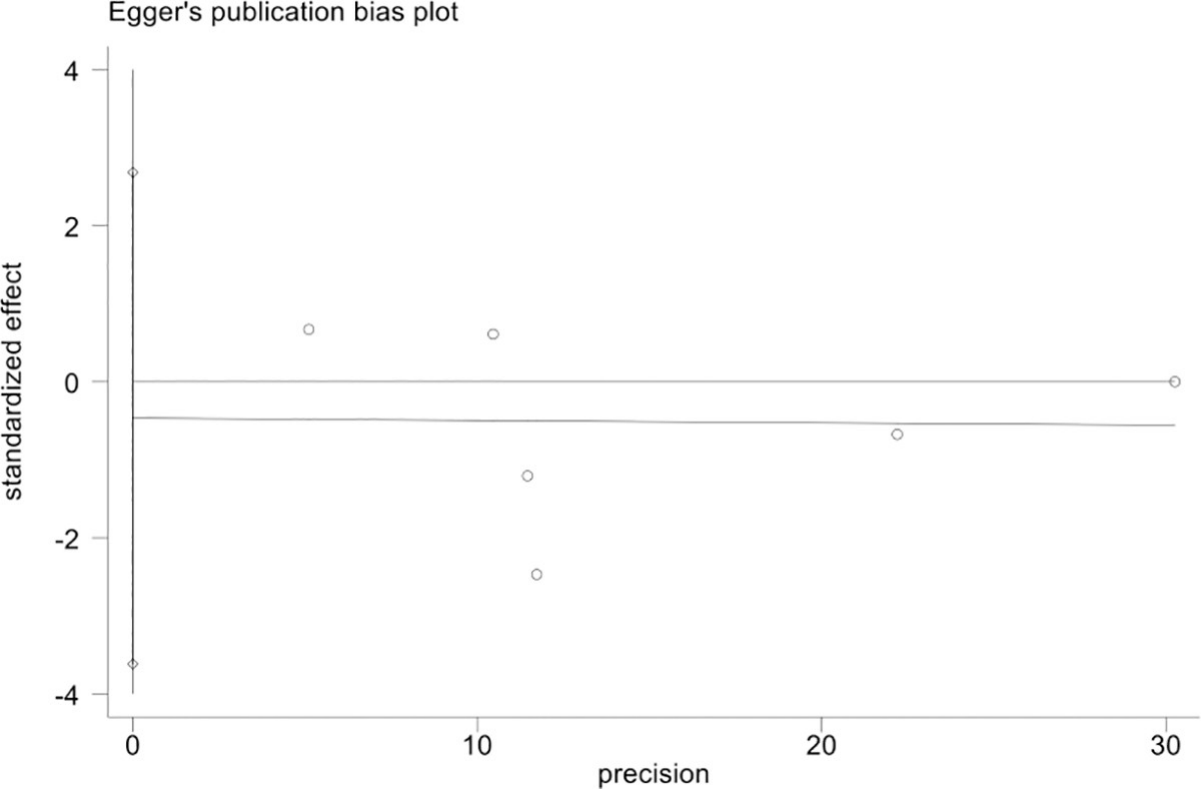
Egger’s publication bias plot.

Publication bias was determined using funnel plots and Egger’s test. The funnel plots for publication bias using the fusion rate in the final follow-up suggest no obvious asymmetry, and the funnel plots indicated that the pooled results were not influenced by the publication bias (Fig 6). An Egger’s linear regression method suggested no statistically significant evidence of publication bias was observed (P = 0.701) (Fig 7).

## Discussion

Increasing the fusion rate, improving pain release and decreasing complications were important in spine fusion surgery and were also the focus of many researchers. Recently, the use of PRP in spinal fusion surgery was investigated, and the results [8], which were not consistent with those of previous studies [9, 16], were promising. Several systematic reviews have evaluated the effectiveness of PRP for spinal fusion. Elder et al. concluded that there is insufficient evidence to recommend the widespread use of PRP in spinal fusion surgery [15]. Park et al. reported that PRP might promote human spinal fusion if the platelet count or the concentration of growth factors in the PRP increases [25]. However, those studies included both animal and clinical studies and did not pool the available data for analysis. Our analysis aimed to include all available prospective studies evaluating the effectiveness and safety of PRP in spinal fusion, and moreover, we addressed whether PRP might increase the fusion rate and better relieve pain with fewer complications. No apparent publication bias was found according to the evaluation of the funnel plot and Egger’s test. To our knowledge, the present results derived from all available comparative data are the first to document the fusion rate, pain and complications between PRP and non-PRP in spinal fusion surgery.

PRP, which contains several growth factors, such as platelet-derived growth factor (PDGF) and transforming growth factor-b (TGF-b) [26, 27], is an autogenous source of growth factors with osteoinductive properties [28, 29]. As previously described, PRP is created by extracting platelets from whole blood and resuspending these factors in a small volume of plasma to form a concentrated platelet solution [5]. Although PRP is widely used in orthopedic, cardiovascular, and dental treatments, there is still a lack of a standard for PRP, including the extraction method, concentration level, activation method, whole blood volume needed and total volume after extraction. Mishra et al. proposed a system that classifies PRP by leukocyte content, platelet number, and activation into 4 types and 2 subtypes [30]. It is important to objectively assess the effectiveness of PRP. However, this system included only some of the factors that can influence PRP, and more details should be considered for classification.

In terms of fusion rate, our study included different methods to evaluate the fusion rate (i.e., CT and X-ray) at various timepoints (12 weeks, 4 months, 6 months, 1 year, 2 years and 10 years). CT scanning is more reliable for investigating bone union than X-ray. Even on flexion–extension films, it is difficult to assess fusion for several reasons: there is a difference in the range of motion of 7 to 14 in asymptomatic individuals, pain may inhibit motion, and the measurement of motion may be unreliable in the presence of pedicle-screw instrumentation [31]. Three of the included studies used only X-ray imaging to assess the fusion rate [16, 20, 23], and three studies used both CT and X-ray imaging for evaluation [8, 18, 22]. Jenis et al. used CT for 6-month evaluations and X-ray for 1- and 2-year evaluations postoperatively. CT was performed at 1 year after surgery for fusion assessment in the Tsai et al. study. These factors may increase the potential heterogeneity of the results. Pooled analysis of the RCTs or observational studies suggested that there was no significant difference between groups. As varied timepoints of fusion rate assessment were reported, we performed a subgroup analysis of RCTs based on different timepoints. The results at both 3 months and 12 months postsurgery showed no significant difference between groups. Interestingly, we discovered that PRP groups may have achieved faster fusion at 6 months after surgery than did the control, although the difference was not significant, and only 2 RCTs reported the fusion rate at this timepoint. Other studies have also revealed a positive effect of PRP on accelerating fusion in a shorter time. Hartment et al. reported that the use of PRP provides faster fusion (8.33 vs. 12.5 months) and higher density values within the fusion mass for patients who sustained a traumatic fracture of the spine. Imagama et al. conducted a study of patients who underwent one-level lumbar fusion, and the local bone on the left side and the local bone treated with PRP on the right side were grafted. The results showed that the bone absorption assessed by CT was significantly lower on the PRP side at 3 and 6 months after surgery. A prospective cohort study performed by Tarantino et al. compared two sides at one-level fusion and demonstrated increased bone density using PRP and a heterologous cancellous block resulting in an enhanced fusion rate during the first 6 months after surgery [32]. According to these results, we considered PRP to have a positive effect on the early fusion of the spine. However, because of the limited data, we could not conclude that PRP treatment would stimulate a shortened time of fusion.

Most of the studies included in our research had a follow-up of one and two years after surgery, which seems to be a short time. Only one study reported a result with a ten-year follow-up [21]. Although some researchers concluded that the fusion rate at one year after surgery did not differ significantly from the rate at two years postoperatively [16], one study considered the same fusion rate at one and two years postsurgery. As we do not know how long PRP can affect the bone and whether it can release growth factors continuously, studies with longer follow-up periods are needed to better assess the effectiveness and safety.

Clinical studies of PRP for low back pain patients have commonly shown that the intradiscal injection of PRP was effective in reducing back pain [33–35]. In our research, five of the included studies provided VAS scores, and all studies showed a significant decrease in pain scores after surgery compared with preoperative scores. However, the effect on relieving pain was comparable, regardless of PRP use. Four studies compared VAS scores between groups after surgery [8, 9, 19, 22]. Consistent with a previous review [5], compared with controls, PRP did not appear to reduce pain.

Regarding safety, the complication rate was consistent with a previous study. Therefore, using PRP in spinal fusion surgery did not increase side effects or complications. The complications reported in the included studies were infection, recurrent nerve palsy, screw protrusion, dural tears, revision and pseudarthrosis. The most common complication was pseudarthrosis. However, a study by Castro et al. reported a much higher complication rate than that in other studies, while the characteristics of the participants, surgical method and PRP concentration did not significantly differ between studies. The main reason for this result might be the lack of agreement on clear definitions of complications [12]. This disagreement makes statistical comparisons difficult. On the other hand, some complications might be ignored and may not be reported due to this lack of agreement. We believe it is meaningful to clinical practice to further assess the safety of PRP.

Although the surgical approaches, including anterior-posterior, posterior, and posterolateral approaches, the fusion method, as well as instrumented and noninstrumented fusion, varied in our included studies, which may be a potential factor influencing the PRP, some studies have concluded that different approaches in spine fusion did not affect the fusion rate [36, 37]. The preparation of PRP greatly varies and may be the cause for the differing biological activities reported among studies. Different PRP activation methods affect its physical form and can also change the release of bioactive molecules [38]. Moreover, as the methods of concentration differed in the various studies, the relationship between the effect of PRP on fusion and the fold-increase in platelets after concentration should be further assessed. A prospective cohort study suggested that no correlation between bone density and preoperative platelet count was observed [39]. However, an opposite result was shown in the Kubota et al. study, in which the average platelet count was 7.7 times higher and the growth factor concentrations were 50 times higher in PRP than in blood plasma, and these factors effectively shortened the time of bone union by approximately 2 months and promoted a higher fusion mass [8]. Another study showed that PRP had no stimulating effect if the PRP platelet count was five times that of peripheral blood [24]. Therefore, based on these limited data, whether there is a linear relationship between platelet count and bone fusion remains unknown. The bone grafts and extenders, including iliac bone, allograft bone and lamina, were also varied in the included studies. Eight of the included studies reported using autografts and two studies reported using allografts in their research. Kubota, Sys et al., Acebral-cortina et al., and Tsai et al. performed fusion using a local bone graft generated from the lamina in two groups. Imagama et al. used milled lamina and spinous process bone as the bone graft in two groups. Hartmann et al. used a bone graft drawn out of the fractured vertebra. Castro et al. used an iliac crest bone graft in both groups. Rezende CF et al. reported using spinal process, laminae, and articular facets in two groups. Feiz-erfan et al. reported using allografts in both groups. Jenis et al. reported using an iliac crest bone graft in the control group and an allograft in the study group. These grafts and extenders may have substantially different effects on spinal fusion outcomes and have previously been shown to have varying effects on bone tissue engineering with PRP [40, 41]. However, the limited data could be analyzed to assess the potential effects of different bone grafts on fusion.

Many factors can influence the rate of spinal fusion, including diabetes mellitus, smoking habits, and alcohol intake, and these factors may have an impact on bone union [21, 42]. Only four of the included studies measured the characteristics of the smoking habits of the participants [9, 19, 21, 23], and this factor might have influenced our findings, as two recent studies have observed a significant association of pseudarthrosis development among smokers with a positive smoking status in anterior lumbar interbody fusion [2, 43].

Interestingly, substantial differences in surgical indications were observed among patients in different studies. Indications in most of the included studies were degenerative disease, herniated disc, spinal stenosis and spondylolytic spondylolisthesis, and all the surgical procedures were primary fusions. Patients with lumbar fracture were included in only one study [19], which suggests a positive effect of PRP on enhancing bony fusion. Another retrospective study involving 20 participants and 5 lumbar fractures suggested that PRP used with a cancellous bone substitute increases the rate of fusion and bone density. Feiz-erfan et al. reported that compared with a cohort of patients with disc herniation, patients with degenerative discs treated with a platelet gel demonstrated early fusion at the 12-week follow-up interval [16]. PRP was proven to have a positive effect on tissue and bone healing [44, 45]. It was speculated that the more extensive bone removal associated with the exposure of the cancellous material needed for decompression in the patients with degenerative disease afforded a better substrate for the platelet concentrate effect [16]. However, limited data were available to further evaluate which surgical indication was suitable for using PRP. Future studies should focus on the effects of PRP for different surgical indications.

### Strengths and limitations

The present results derived from all the available prospective comparative data are the first to document the differences in the postoperative fusion rate, pain release, and complications between PRP and non-PRP treatments in spine fusion. Our study has several limitations. First, most of the included studies had low methodological quality. Only 3 RCTs and 7 observational studies with a small sample size were included, publication bias tests might be underpowered to detect the significance, and sample size heterogeneities might have biased the meta-analysis. Second, a standard concentration method, concentration, volume and activation of PRP, which were not consistent in the included studies, was lacking and may have increased the potential bias of the pooled analysis results. Third, in addition to the different surgical indications, such as spinal fracture, degenerated discs, spinal stenosis, and spinal instability, which were included in the present study, other parameters, such as the surgical approach, the graft and extender, rehabilitation program, and smoking habits, which may influence bone union progress after surgery, varied in the studies; previous studies noted that these factors were important for the prognosis of spine fusion. Fourth, the method of assessing fusion after surgery was not consistent, as some studies were assessed by plain radiographs while other studies were assessed by CT. Moreover, although the details of the complications were measured in some of the included studies, the details of the complications in other studies remain unknown, and because of these limited data, we only evaluated complication rates and pseudarthrosis, which were measured in the included studies. Despite these limitations, the present systematic review and meta-analysis demonstrated that using PRP in spine fusion might accelerate early bone union and reduce pain but may not increase the fusion rate or complication rate.

## Conclusion

In conclusion, PRP used in spinal fusion surgery increases neither the fusion rates, clinical outcome nor complication rates. However, this treatment might accelerate early bone union postoperatively. Additionally, PRP might increase the fusion rate of patients with spinal fractures. Therefore, based on cost effectiveness, regular use of PRP should not be considered for spinal fusion surgery. However, because only a few RCTs with small sample sizes and varied PRP preparations were included in the present study, more high-quality RCTs are needed to further evaluate the effect of PRP and the diseases appropriate for its use.

## Supporting information

S1 Checklist PRISMA 2009 checklist. (DOC)

S1 Table Search strategies in Pubmed. (DOC)
