# Supplementary material for: The effect of platelet-rich plasma on the fusion rate and clinical outcome of spinal fusion surgery: A systematic review and meta-analysis
Source: PLoS One. 2020 Dec 3;15(12):e0243204. doi: 10.1371/journal.pone.0243204 (PMC7714170; doi:10.1371/journal.pone.0243204)
Supplement: S1 Table — (DOC) [file pone.0243204.s002.doc]

S1 Table. Search strategies in Pubmed.

#1 Spinal fusion[MeSH Terms]

#2 Fusion, Spinal[Title/Abstract]

#3 Fusions, Spinal[Title/Abstract]

#4 Spinal Fusions[Title/Abstract]

#5 Spondylodesis[Title/Abstract]

#6 Spondylodeses[Title/Abstract]

#7 Spondylosyndesis[Title/Abstract]

#8 Spondylosyndeses[Title/Abstract]

#9 Spinal arthrodesis[Title/Abstract]

#10 Lumbar arthrodesis[Title/Abstract]

#11 Lumbar Interbody Fusion[Title/Abstract]

#12 #1 OR #2 OR #3 OR #4 OR #5 OR #6 OR #7 OR #8 OR #9 OR #10 OR #11

#13 plasma, platelet rich[MeSH Terms]

#14 platelet-rich plasma[Title/Abstract]

#15 platelet glue[Title/Abstract]

#16 PRP[Title/Abstract]

#17 platelet gel[Title/Abstract]

#18 platelet fibrin[Title/Abstract]

#19 #13 OR #14 OR #15 OR #16 OR #17 OR #18

#20 #12 AND #19
